# The Role of Medical Students in the Fight to Control Neglected Tropical Diseases: A View from Peru

**DOI:** 10.1371/journal.pntd.0000292

**Published:** 2008-09-24

**Authors:** Javier Villafuerte-Galvez, Walter H. Curioso, J. Jaime Miranda

**Affiliations:** 1 Scientific Society of Medical Students (“SOCEMCH”), “Alberto Hurtado” School of Medicine, Universidad Peruana Cayetano Heredia, Lima, Peru; 2 School of Medicine, Universidad Peruana Cayetano Heredia, Lima, Peru; 3 School of Public Health and Administration, Universidad Peruana Cayetano Heredia, Lima, Peru; 4 Department of Medical Education and Biomedical Informatics, School of Medicine, University of Washington Seattle, Washington, United States of America; 5 Department of Epidemiology and Population Health, London School of Hygiene and Tropical Medicine, London, United Kingdom; New York Blood Center, United States of America

## Introduction

Neglected tropical diseases (NTDs) is a term that might not ring a bell among the majority of our fellow medical students. The World Health Organization (WHO) defines NTDs through a list of 15 diseases, all of them infectious, ancient, and debilitating [1]. Despite vast consensus on which are and which are not considered NTDs, the precise inclusion criteria are as hard to define as global health [2]. Nevertheless, we believe that three basic features that characterize NTDs are high burden of disease in certain specific contexts, neglected from prevention and control—including drug development, and long-term impairment among those suffering from them. Neglect is the central idea, because not only do these diseases sicken the historically neglected populations, but they also have long been neglected from the axis of research, innovation, and production. The objective of this essay is to describe and promote training opportunities on NTDs to medical students, especially in Peru and Latin America. We will describe two medical student organizations and how they are organized to address NTDs. Finally, we will suggest three methods—curriculum, research, and information dissemination—of raising awareness of NTDs among medical students in Peru.

Defining the NTDs through globally applicable and locally sensible criteria, instead of a rigid pre-specified list, could eventually improve the struggle against them. In this effort, medical students should play an important role. The Peruvian National Institute of Health, by establishing long-neglected diseases specific to Peru—such as Carrión disease due to *Bartonella bacilliformis* [3] and hydatidosis [4]—as national public health priorities for research, has shown the imperative of the need for a local NTD agenda [5].

## How Are Medical Students Contributing to the Fight against NTDs?

Medical students worldwide have been exposed to NTDs in different ways. In Peru, from the WHO's list of the main 15 NTDs, some of them could be deemed familiar to the medical students, i.e., trachoma, leishmaniasis, Chagas disease, soil-transmitted helminths, and leprosy. In addition, medical students who took part in extracurricular clinical, research, or community activities might be exposed to cholera/epidemic diarrheal diseases and dengue/dengue hemorrhagic fever.

Some of the NTD-related activities where medical students have been involved worldwide and, specifically in Peru, include the following:

### 

#### The role of medical students' organizations

##### The IFMSA model

The International Federation of Medical Students' Associations (IFMSA) has been organizing projects and workshops, establishing networks, and promoting medical students' exchanges for many years. Most of these activities are related to global health issues (including NTDs). The organization is run entirely by the students themselves, including all the necessary fundraising. Revealing a strong desire to improve global health, some students have actively sought training to understand and participate in the international debate [6]. In Peru, the Medical Students' Scientific Society at Universidad Peruana Cayetano Heredia (SOCEMCH) has been collaborating with IFMSA for nearly eight years [7]. In the last four years, through SOCEMCH, foreign students from Spain, Brazil, Germany, and Canada have visited Peru and joined in projects related to NTDs such as leishmaniasis and bartonellosis. These experiences, although probably valuable at the individual level, have limited impact on the life of people burdened by NTDs.

##### The Latin American SOCEM model

During the 1990s, motivated groups of medical students from individual medical schools across Latin America founded Medical Students' Scientific Societies (Sociedades Científicas de Estudiantes de Medicina [SOCEM]), which eventually federated into national and regional organizations. Examples of these organizations are the Peruvian Medical Students' Scientific Society (SOCIMEP) and the Latin American Federation of Medical Students' Scientific Societies (FELSOCEM) [8]. These SOCEM-like organizations are structured to congregate and stimulate medical students to work in three main areas: research, education, and community intervention. This could be an ideal platform for involving medical students on a larger regional scale in the battle against NTDs. There have been numerous and commendable short-term efforts by these organizations towards that direction. Some outputs include the organization of a yearly international congress for Latin American students and the publication of CIMEL, a journal entirely run by medical students (see http://www.scielo.org.pe/scielo.php/script_sci_serial/pid_1680-8398/lng_es/nrm_iso).

At Universidad Peruana Cayetano Heredia (UPCH), the medical students' SOCEMCH [9] has the mission to promote research at the undergraduate level. SOCEMCH has also been organizing public health activities since 1992. Medical students turn the passive role of getting information into an active role of learning with the community. The application of research and community participation constitutes an effective way of learning. For example, in 1999 as part of an outreach community activity, medical students from SOCEMCH along with UPCH faculty and biologists visited a rural Andean community in Huanuco to conduct research and community service related to leishmaniasis and intestinal parasitosis [10],[11],[12].

Sustainability of many projects at both FELSOCEM and SOCEMCH is built on existing projects, i.e., grants funded by national or international institutions, although many others are self-funded out-of-pocket by students themselves.

## What About Those Students Who Are Not Part of Established Students' Organizations?

As Gavin Yamey has rightly pointed out, “one problem facing the community working on controlling NTDs is the lack of communication between the various players—researchers, policymakers, clinicians, public-private partnerships, donors, and patient advocacy groups” [13]. This disarticulation unfortunately includes medical students. Nevertheless, as they will be the future clinicians, researchers, and leaders of organizations, they should constitute one of the ideal targets for any NTD sensitization strategy.

Kishore et al. [14] has shown that it is possible for medical students in the US to lead a movement that has rapidly gathered researchers, more students, and different organizations to tackle NTDs. In the case of Peru, a country with tropical areas, medical students have, to a certain degree, higher chances than others of being exposed to NTDs—either through lectures, laboratory sessions, or patient care. However, such exposure does not entail the term NTDs nor its underlying concept. The term neglected underlines the importance of poverty, exclusion, and lack of attention to such diseases—a framework that is not pointed out very often during regular training.

Nevertheless, isolated successful experiences could amalgamate into making progress on NTDs at the student level in Peru. These experiences go along the lines of 1) training and curricular change, 2) research, and 3) dissemination.

### 

#### Sensitization and curricular change

As part of training, and using the framework of a global health training program, the Global Health Peru Program at UPCH (http://www.globalhealthperu.org) has included approximately three hours of NTDs in their annual course “Basic Concepts in Global Health.” This two-credit, intensive one-week course has been offered since 2006 and is directed at undergraduate students [15]. It is envisioned that this course will be incorporated within medical school curriculum, and the inclusion on NTDs as part of its core components provides a strategic step towards the inclusion of NTDs into broader training. SOCEMCH's Standing Committee on Public Health has made NTD sensitization a priority for 2008 and the years to come.

#### Research careers forged since medical school

Using established research groups in broader infectious diseases, the incorporation of medical students at earlier stages has been crucial in influencing research careers after medical training. For example, taking advantage of intestinal parasitic research, Marcos and Maco have started to make important scientific contributions to human fascioliasis, another infectious disease of importance to Peru [16],[17]. In the same vein, Miranda-Verastegui in leishmaniasis [18], Huarcaya in bartonellosis [19],[20], Miranda in cholera [21],[22], and Bustos in cysticercosis [23],[24] have made important contributions to research on Peru-specific NTDs. All of them share the experience of an early exposure, as medical students, to research in not-so-common diseases, taking advantage of a research infrastructure already in place as well as mentorship from established researchers in their fields. For example, following graduation, Miranda-Verastegui and Bustos have started successful research careers on leishmaniasis and bartonellosis.

#### How should we disseminate that research?

As for dissemination, this area looks very promising for medical students thanks to the advantage of the scientific open-access movement.

The CIMEL journal [25], the official voice of FELSOCEM and indexed in SciELO, is entirely edited by medical students from Latin America and was created as an alternative to disseminate research and foster publication amongst undergraduate medical students [26]. Some interesting articles related to NTDs have already been published in CIMEL by medical students. The next step for CIMEL would be to increase its global exposure, an endeavor that can be particularly challenging without PubMed indexation. A barrier for Latin American students may be writing in English, which could be considered a time-consuming and difficult enterprise for some. Nevertheless, a growing number of open-access channels with widespread exposure, such as the Public Library of Science and BioMed Central journals, are already in place. These initiatives will also directly benefit medical students who would be using these resources more actively. In addition, medical students have published in Peruvian open-access journals about NTDs such as *Revista Médica Herediana* [27], *Revista de Gastroenterología del Perú* [28], and *Acta Médica Peruana* [29].

## Is That Enough?

Most of the medical student research in Peru—including NTDs research—is descriptive and, although necessary, is not yet enough to produce effective and efficient interventions or technologies that could have impact on people with NTDs. Efforts in that direction should be energetically encouraged and financially supported. The few initiatives already developed by medical students require resources and political support from academic, public, and private organizations to secure progress in the field.

## Conclusion

NTDs are a group of conditions that should matter to medical students because of their impact in the most neglected sectors of society. Nonetheless, the global discourse of NTDs requires local nuances to make the message relevant to specific contexts. By taking advantage of organized medical students' structures, or shaping ongoing activities in training, research, and publication, we have the potential to develop a more cohesive and stronger message about NTDs among Peruvian medical students.

## Supporting Information

Alternative Language Abstract S1 Translation of the Abstract into Spanish by Javier Villafuerte-Galvez, Walter H. Curioso, and J. Jaime Miranda
(0.02 MB DOC)
