# Supplementary material for: The Role of Medical Students in the Fight to Control Neglected Tropical Diseases: A View from Peru
Source: PLoS Negl Trop Dis. 2008 Sep 24;2(9):e292. doi: 10.1371/journal.pntd.0000292 (PMC2561065; doi:10.1371/journal.pntd.0000292)
Supplement: Alternative Language Abstract S1 — Translation of the Abstract into Spanish by Javier Villafuerte-Galvez, Walter H. Curioso, and J. Jaime Miranda (0.02 MB DOC) [file pntd.0000292.s001.doc]

## Resumen

Los estudiantes de medicina pueden jugar un rol importante en la lucha contra las enfermedades tropicales desatendidas (ETD), en particular en los países en desarrollo. Basándonos en experiencias de nuestro entrenamiento médico actual y pasado, este ensayo explora oportunidades de entrenamiento u otras disponibles para estudiantes de medicina, especialmente en Perú y Latinoamérica. Especificamente, dos organizaciones de estudiantes de medicina y su trabjajo relacionado a ETD son analizadas. Finalmente, los autores sugieren tres métodos –currícula, investigación y diseminación de información— e historias exitosas para fomentar el tema de las ETD entre estudiantes de medicina dentro y fuera del Perú. Las ETD pueden llegar a influir de manera más sólida y coherente entre los estudiantes de medicina aprovechando estructuras ya establecidas o integrándose en actividades de entrenamiento, investigación y publicación.
